# RACIAL DIFFERENCES IN ADVANCE CARE PLANNING IN SAN ANTONIO, TEXAS

**DOI:** 10.1093/geroni/igae098.3203

**Published:** 2024-12-31

**Authors:** Jonathan Dao, Cory Kittleman, Pratima Gangupantula, Ninette Siby, Avinash Kakulavar, Rhea Gogia, Jason Morrow

**Affiliations:** The University of Texas Health Science Center San Antonio, San Antonio, Texas, United States; The University of Texas Health Science Center San Antonio, San Antonio, Texas, United States; The University of Texas Health Science Center San Antonio, San Antonio, Texas, United States; The University of Texas Health Science Center San Antonio, San Antonio, Texas, United States; The University of Texas Health Science Center San Antonio, San Antonio, Texas, United States; The University of Texas Health Science Center San Antonio, San Antonio, Texas, United States; The University of Texas Health Science Center San Antonio, San Antonio, Texas, United States

## Abstract

Advance directives (AD) convey patient’s preferences about end-of-life care to physicians and loved ones when they lose capacity. Only 45.6% of older adults (OA) have filled out an advance directive due to lack of time, knowledge, or comfort discussing the topic. Our organization prepared and delivered presentations on ADs at seven community centers in San Antonio, Texas to primarily caregivers, community health workers, and OA. Pre- and post-surveys were developed to assess demographics and evaluate attitudes (Likert scale) regarding ADs. Of 119 surveys, 50 respondents were OAs over 65 years old with a mean age of 74.8; 48% (n=24) identified as Hispanic/Latino, 30% (n=15) as African American (AA), and 25% (n=13) as White. 37.5% of Hispanic, 40% of AA, and 61.5% of White respondents reported previously completing an AD. 29.17% of Hispanic, 46.7% of AA, and 69.2% of White respondents reported previously completing an MPOA. 37.5% of Hispanic, 29.7% of AA, and 46.2% of White respondents reported being offered help from a physician to complete an AD. A higher proportion of White OAs had completed an MPOA, AD, and were offered help by a physician than AA or Hispanic/Latino OAs. This data remains consistent with previous studies on racial disparities in AD completion. A majority of respondents (68%) were never offered help from a doctor to complete an AD indicating a need to continue engaging patients, especially those of lower socioeconomic status.

